# Vesicle-based formulations for pain treatment: a narrative review

**DOI:** 10.1097/PR9.0000000000001196

**Published:** 2024-10-10

**Authors:** Juan Martinez, Nicola Ingram, Nikil Kapur, David G. Jayne, Paul A. Beales

**Affiliations:** a School of Chemistry, University of Leeds, Leeds, West Yorkshire, United Kingdom; b Leeds Institute for Medical Research, University of Leeds, Leeds, West Yorkshire, United Kingdom; c School of Mechanical Engineering, University of Leeds, Leeds, West Yorkshire, United Kingdom; d The John Goligher Colorectal Surgery Unit, St. James's University Hospital, Leeds Teaching Hospital Trust, Leeds, West Yorkshire, United Kingdom

**Keywords:** Pain relief, Vesicle-based technologies, Drug delivery, Analgesics, Liposomes, Polymersomes, Ethosomes, Niosomes

## Abstract

Pain, a complex and debilitating condition, necessitates innovative therapeutic strategies to alleviate suffering and enhance patients' quality of life. Vesicular systems hold the potential to enhance precision of drug localisation and release, prolong the duration of therapeutic action and mitigate adverse events associated with long-term pharmacotherapy. This review critically assesses the current state-of-the-art in vesicle-based formulations (liposomes, polymersomes, ethosomes, and niosomes) for pain management applications. We highlight formulation engineering strategies used to optimise drug pharmacokinetics, present preclinical findings of experimental delivery systems, and discuss the clinical evidence for the benefits of clinically approved formulations. We present the challenges and outlook for future improvements in long-acting anaesthetic and analgesic formulation development.

## Abstract 2

Vesicle-based drug delivery offers promising solutions by enhancing efficacy, reducing toxicity, enabling targeted pain relief, and overcoming the limitations of current treatments.

## 1. Introduction

**Figure 1.**
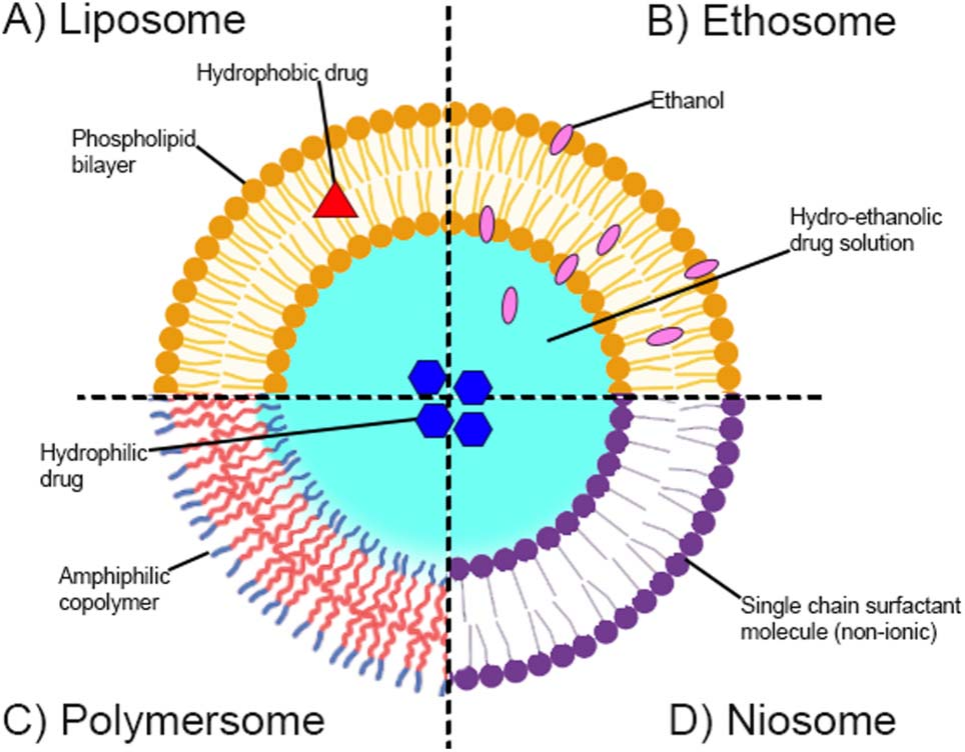
Typical vesicle-based nanocarriers that are used for the treatment of pain. (A) A liposome composed of a phospholipid bilayer. (B) An ethosome made of a phospholipid bilayer and alcohol (ethanol is commonly used). For drug encapsulation, a hydroethanolic drug solution must be used. (C) A polymersome formed by amphiphilic copolymers. (D) A niosome made up of nonionic single-chain surfactants. All these different types of vesicles can incorporate a hydrophobic drug inside the membrane or a hydrophilic drug in the aqueous lumen.

Pain is a complex and multifactorial condition affecting millions worldwide. Despite the availability of numerous analgesic drugs and treatment modalities, many patients still suffer from inadequate pain relief or experience undesirable side effects.^34^ As such, there is a significant need for novel and effective pain management strategies to overcome the limitations of current pain treatments. Vesicle-based technologies are promising drug delivery systems with great potential for treating pain. These systems are composed of small (nanometre to micrometre scale). These spherical structures can encapsulate a variety of therapeutic agents, such as opioids, nonsteroidal anti-inflammatory drugs (NSAIDs), and local anaesthetics.^24^ They are designed to enhance drug efficacy, reduce toxicity, and improve patient compliance, among other benefits.^46^ The most common vesicle-based nanocarriers are liposomes, polymersomes, ethosomes, and niosomes (Fig. 1).

Liposomes are made of a phospholipid bilayer. The phospholipids comprise a hydrophilic head group and (usually 2) hydrophobic tails. These phospholipids spontaneously self-assemble in an aqueous medium, arranging into a double-layer structure that protects the hydrophobic tails from energetically unfavourable contact with water molecules. These bilayers wrap into a spherical membrane shell to form the hollow vesicle structure with an internal aqueous lumen. Liposomes can be unilamellar (one bilayer membrane) or multilamellar, where many bilayer membranes often form a concentric onion-like structure.^80^

Polymersomes are similar in structure to liposomes but made of amphiphilic block copolymers instead of phospholipids.^18^ A wide range of amphiphilic block copolymer structures have been demonstrated to self-assemble into vesicle structures, the simplest being linear diblock copolymers, where a hydrophilic polymer and a hydrophobic polymer are covalently linked. However, more complex multi-block polymers and polymers with nonlinear, branched architectures have also been shown to form vesicles. These polymer vesicles generally take longer to release their contents than liposomes and can be designed to have a higher drug-loading capacity. This is because of the higher molecular weight of block copolymers compared with phospholipids, and so polymersomes tend to have a thicker membrane (∼5–50 nm) than liposomes (∼3–5 nm), and the longer polymers often entangle in the membrane, giving greater structural cohesion. It is important to point out that although both polymersomes and lipid-based vesicles use passive diffusion as the primary mechanism for drug release, biodegradable polymersomes can experience matrix erosion,^44^ and they are overall easier to chemically modify to create stimuli-responsive vesicles^66^ compared with lipids.

Ethosomes are made of phospholipids and alcohol, typically ethanol. Adding ethanol to the membrane makes it more permeable, making it a preferred choice when applying the formulation through the skin.^81^ Niosomes are vesicles made of single-chain nonionic surfactants, forming a bilayer similar to liposomes. However, they lack the capacity for targeted drug delivery.^25^

**Figure 2.**
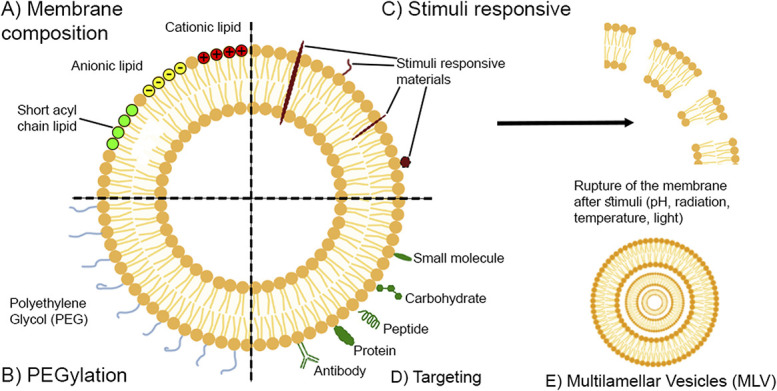
Vesicle engineering for pain treatment. (A) The composition of the membrane can be tuned using different types of lipids such as cationic, anionic, or short acyl chain. (B) Multiple stimuli-responsive materials can be incorporated into the membrane. In most cases, after the addition of the appropriate stimulus, the membrane ruptures for a triggered release. (C) PEGylation is a common technique for developing vesicles with long circulation times. (D) Different receptor-specific ligands can be engineered into the membrane for more targeted drug delivery. (E) Liposomes can be designed to possess multiple concentric aqueous compartments instead of one. These are called MLV. MLV, multilamellar vesicles.

Vesicle-based technologies can improve drug bioavailability, reduce toxicity, and provide targeted drug delivery to specific tissues or cells,^74^ making them a highly attractive option for pain treatment. Considerable research in this field has yielded different types of vesicle-based systems that can be engineered to control their stability, size, and drug-loading capacity.^23^ There are 2 main types of modifications for pain treatment: the composition of the membrane and surface modifications (Fig. 2).

Different lipids, or surfactants, can control the physical properties of the vesicles, such as the membrane fluidity, charge, stability, and drug loading capacities.^79^ For example, using lipids with shorter acyl chains can increase the fluidity of the vesicle membrane, making it more permeable to the encapsulated drug. By contrast, using lipids with longer acyl chains can result in a more rigid and less permeable membrane^79^ (Fig. 2A). Similarly, the degree of unsaturation of the hydrocarbon acyl chains affects the membrane's fluidity, permeability, and stability. Unsaturated cis double-bonds in the acyl chain create “kinks” in these tails that disrupt the ordered packing of the lipids, making the membranes more loosely packed and fluid^87^ (Fig. 2A). Saturated lipids are more commonly used in vesicle nanomedicines to enhance the stability and lower the membrane's permeability for long-acting parenteral delivery applications.^6^ However, saturated lipids alone tend to form rigid, solid-like membranes because of their ordered packing into gel phases.^6,87^ Therefore, saturated lipids are combined with cholesterol (Chol), which fluidises the membrane into a liquid-ordered state that maintains a tightly packed membrane structure but can accommodate the curvature required to bend into spherical capsules without packing defects that can compromise the vesicle stability and permeability.^61^

Cationic surfactants can create positively charged vesicles, which are attracted to the negatively charged cell membranes, facilitating their uptake. Alternatively, anionic surfactants can form negatively charged vesicles that interact less with the cellular membrane. Nonionic surfactants are less charged and can create a more stable vesicle than anionic and cationic surfactants, prolonging their shelf life^62,80^ (Fig. 2A).

Surface modifications have been shown to improve the efficiency and effectiveness of pain management by providing targeted transport to specific tissues or cells. Ligands such as antibodies or peptides that recognise receptors on the cell surface and targeting moieties such as aptamers or sugars that selectively bind to tissues or organs can be used for these modifications.^69^ Common cell-surface receptors for pain management include ion channel-linked receptors, G protein–coupled receptors (GPCRs) and tyrosine kinase–linked receptors, which are well-known receptors that trigger an intracellular signal and response to harmful stimuli.^48^ In addition, the addition of polyethylene glycol (PEG), or other inert, hydrophilic “anti-fouling” polymers, to the surface of vesicles can enhance their stability and reduce their clearance rate from the body by creating a “stealth” coating at the particle surface, thus prolonging their circulation time and increasing drug accumulation in the target site (Fig. 2B). This process, known as PEGylation, is often selected when long circulation times are required.^4,77^

Another approach to enhancing vesicle functionality is to modify the vesicle surface with stimuli-responsive materials (Fig. 2C). These materials respond to specific external stimuli, such as pH, temperature, light, or magnetic fields.^56^ pH and temperature are key local environmental changes during inflammation (and hence pain), both because of the activation of inflammatory cells in affected tissues.^13,64^ The versatility of functional adjustments possible for vesicle structures enables the creation and customisation of tailored formulations designed to suit the precise requirements of individual therapeutic uses.^43^

For example, opioids are commonly used for pain management but have a high risk of addiction and respiratory depression.^38^ Vesicles can be used to encapsulate opioids and deliver them in a targeted manner, reducing the negative side effects.^16^ Local anaesthetics, such as lidocaine and bupivacaine, are commonly used to manage acute and chronic pain. Still, the effects of current formulations are short-lived and may require multiple injections, leading to complications such as nerve damage.^12^ A vesicle-encapsulated version of these drugs could improve the pharmacokinetics of local anaesthetics by increasing their half-life through slow liberation of the active drug molecule and reducing their systemic toxicity, allowing for more sustained pain relief.^32^

In addition, the blood–brain barrier (BBB) poses a significant challenge in developing effective pain therapies. The BBB is a highly selective membrane that separates the central nervous system (CNS) from the rest of the body, limiting the entry of many drugs into the brain.^11^ However, vesicle-based technologies can overcome this barrier by encapsulating drugs within liposomes, polymersomes, or other vesicles. These vesicles can then be engineered to target specific receptors on the BBB or neurons in the CNS, allowing for the delivery of therapeutic agents to the brain.^11^

Vesicle-based technologies also offer a promising approach to treating neuropathic pain, a type of chronic pain that arises from damage to or dysfunction of the nervous system. Neuropathic pain is notoriously difficult to treat, and current therapies often provide only partial relief. However, vesicles can be engineered to target the specific mechanisms underlying neuropathic pain, such as the hyperexcitability of neurons, inflammation, and changes in ion channels (eg, upregulation of Na^+^ channels)^22^ (Fig. 2D). Encapsulating drugs with low therapeutic effects for neuropathic pain (when the delivery is not localised) and targeting them to surface receptors involved in neuropathic pain pathways, such as ion channels, has the potential to offer more effective pain relief by enabling a higher concentration of the drug at the pain site.^88^

Overall, using vesicle-based technologies to treat pain holds great promise for improving the efficacy and safety of pain management. By encapsulating drugs within vesicles and engineering them to target specific receptors, it is possible to deliver drugs in a more targeted and sustained way, reducing the risk of systemic toxicity and side effects.^60^ In the following sections, we will review recent advances in vesicle-based technologies for the treatment of pain, with a focus on liposomes, polymersomes, ethosomes, and niosomes. We will discuss the advantages and limitations of each technology and the progress made in preclinical and clinical studies.

## 2. Methods

A comprehensive literature search was conducted to identify relevant articles, reviews, and patents related to the development of vesicle-based formulations for the treatment of pain. Electronic databases, including PubMed/MEDLINE, Scopus, Web of Science, and Google Scholar, were systematically searched using keywords such as “vesicles,” “liposomes,” “polymersomes,” “ethosomes,” “niosomes,” “pain management,” “analgesia,” and related terms. The search aimed to encompass studies published within the last 10 years.

Articles were included in this narrative review based on specific inclusion and exclusion criteria, as detailed in Table 1. These criteria were designed to ensure the relevance and quality of the included studies.

**Table 1:** Inclusion and exclusion criteria for literature selection.

Inclusion criteria	Exclusion criteria
Published in peer-reviewed journals indexed in PubMed, Scopus, or Web of Science	Non–peer-reviewed articles
Published within the last 10 y (2014–2024)	Published within the last 10 y
Focus on vesicle-based formulations for pain management	Studies on non–vesicle-based drug delivery systems
Original research articles (in vitro*,* in vivo*,* clinical studies)	Studies unrelated to pain management or analgesia
Patents related to vesicle-based formulations for pain management	Patents unrelated to vesicle-based pain management
English language publications	Non-English language publications
Clear methodology and well-reported outcomes	Studies with unclear methods or poorly reported results

Given the narrative nature of this review, formal quality assessment tools were not used. Instead, the credibility and relevance of each source were assessed based on the reputation of the publishing venue, author expertise, methodological rigour, and direct relevance to vesicle-based formulations for pain management.

It is essential to acknowledge the potential limitations inherent in narrative reviews. These may include subjectivity in article selection, the possibility of overlooking relevant literature and the absence of a formal quality assessment process. However, these limitations are mitigated by the review's focus on providing a broad understanding and synthesis of existing knowledge on vesicle-based formulations for pain management, supported by explicit inclusion and exclusion criteria to enhance transparency in the literature selection process.

## 3. Results

Through the literature search, 85 articles were identified, of which 34 met the inclusion criteria for this narrative review. The included studies span various publication types, including original research articles with in vivo, ex vivo, and in vitro studies and 2 patents: Probudur and Exparel. The latter has multiple clinical studies that assess its efficacy against currently used pain relief strategies, which were included in this review. These studies collectively provide insights into developing, characterising, and applying vesicle-based formulations for pain management.

## 4. Discussion

### 4.1. Liposomal formulations for pain treatment

The use of liposomes to deliver different drugs is a popular area of research and has expanded over the last 30 years. Liposomal formulations can be tailored by tuning size, composition, and surface morphology to meet therapeutic applications. Various preclinical investigations have been performed involving the encapsulation of different pain-controlling drugs. These studies have utilised a wide range of liposomal compositions and testing methods, summarised in Table 2. One thing consistent across such studies is the reported increased anaesthetic effects and bioavailability of the encapsulated drugs compared with their standard, unencapsulated clinical formulation.

**Table 2:** Preclinical studies of newly developed liposomal vesicles.

Application	Drug encapsulated, efficiency and method	Vesicle composition	Experimental setup (in vivo unless otherwise stated)	Results	Ref.
General chronic pain	Morphine 17.88 ± 6.2% compared with initial drug Passive encapsulation	LUVs: HSPC:Chol:mPEG-DSPE Molar ratio: 56.4:38.3:5.3 MLV: HSPC:CHOL Molar ratio: 2:1	Hot plate test in adult male Swiss mice Conditioned place preference (CPP): assessing drug, liposomal formulation, or control drug dependency	Liposomal morphine persisted for 24 hours postinjection, whereas free morphine was cleared in 5 hours. Liposomal delivery exhibited sustained release in LUV and MLV, with LUV-treated mice showing reduced opioid dependence.	26
Peripheral inflammatory pain	Loperamide HCl 3.71 ± 0.16 mg drug/mL liposome Passive encapsulation	EPC:Chol Molar ratio: 2:1 0.5% wt/wt gel	Paw volume in male Wistar rats. CFA-induced inflammation.	The gel formulations reduced the paw inflammation compared with controls after 48 hours of twice-daily application.	35
Rheumatoid arthritis (RA)	Celecoxib From 90.04 ± 0.3 to 96.6 ± 0.05% compared with initial drug Passive encapsulation	Lipova E120:Chol: MPEG-DSPE_2000_ Molar ratio: 9:1:0.25 9:1:0.5 9:1:1	Paw volume in male Wistar rats. Caragenaan-induced inflammation.	Three formulations released 80% within 72 hours in vitro, and the most efficient was tested in vivo. At 10 mg/kg, celecoxib-loaded liposomes reduced paw volume from 15 to 90 minutes, and 20 mg/kg inhibited oedema compared with pure celecoxib.	85
Local analgesic	Bupivacaine Loading efficiency not mentioned Remote loading	Hydrogenated soy phosphatidylcholine (l-α-PC):CHOL Molar ratio: 2:1	Small clinical trial (n = 6): Assessing analgesia using pinprick postinjection.	MLV formulations provided extended analgesia (up to 48 hours) compared with free bupivacaine (1 hour).	27
Knee osteoarthritis (KO)	6-MNA 72.55 ± 4.25% compared with initial drug Passive encapsulation	6-MNA-DSPE:HSPC:DOTAP:Chol Molar ratio: Not mentioned	Paw volume in male Sprague-Dawley rats. CFA-induced inflammation.	The fortified liposome reduced paw edema by 10% over 14 d compared with free 6-MNA. By day 21, the reduction was 20% less than free 6-MNA. Dose: 4.27 mg/kg.	63
Nociceptive pain	Diflunisal 65.99 ± 1.22% compared with initial drug Passive encapsulation	100% l-α-PC 5% (wt/wt) CMC gel	Paw volume in adult male Wister albino rats. Carrageenan-induced inflammation.	Within 2 hours, the control group's paw swelling sharply increased from 60.86% to 84.38%. Diflunisal hydrogel decreased edema from 66.59% to 30.10% after 2 and 4 h, whereas liposome hydrogels reduced inflammation from 50.10% to 32.53% within the same timeframe.	1
Sciatic nerve blockade (SNB)	N-methyl bromide of lidocaine (QX-314) Efficiency not mentioned Passive encapsulation	l-α-PC:Chol:CHEMS-PEG Molar ratio: 40/17/3	Sensory and motor function using hot-plate test in Sprague-Dawley rats.	QX-314 with liposomes created >24 h sciatic nerve blocks in rats, without systemic toxicity or local tissue injury, surpassing bupivacaine's <5 h of duration.	89
SNB	Tetrodotoxin (TTX) 24% compared with initial drug Passive encapsulation	DSPC:DLPC:DSPG:Chol 0.45 mol% PS Molar ratio: 3:3:2:3	Sensory and motor function using hot plate test in Sprague-Dawley rats.	Injection of light-triggerable liposomes in rat sciatic nerves induced a 13.5 ± 3.1-h of nerve blockade. NIR irradiation 24 h later produced another 2.8 ± 0.9-h block. Liposomes without PS had a 14.6 ± 6.8-h block, with no secondary blockade upon irradiation.	68
Anti-inflammatory	Diacylglycerol lipase-beta (DAGLβ) inhibitor KT109 ∼0.4% Passive encapsulation	DSPC:DOPE:DOPE-PEG_2000_ Molar ratio: 56.6:28.7:14.7	In vitro*:* Macrophage recruitment using thioglycolate in the peritoneal cavity, followed by treatment with test solutions.	Liposomal delivery reduced total KT109 needed for DAGLβ inactivation by over 20-fold compared with free drug.	72
Acute and chronic pain	Opiorphin 69.2 ± 1.2% compared with initial drug Passive encapsulation	l-α-PC: PEG_2000_–DSPE Molar ratio: Not mentioned	Tail-flick latency test in adult male Sprague-Dawley rats	Opiorphin-loaded PEGylated liposomes maintained 98% maximum possible effect (MPE) at 30 min, gradually decreasing to 50% MPE at 60 min. Free Opiorphin reached 60% MPE at 10 min and decreased to 18% after 1 h.	55
Neuropathic pain	Capsaicin 81.9 ± 2.43% compared with initial drug Passive encapsulation	l-α-PC:Chol Molar ratio: Not mentioned	Formulation effectiveness was not assessed; only pharmacokinetic parameters were provided.	At 90 mg/kg dosage, peak plasma concentration increased from 808.2 ± 23 to 864.9 ± 9.77 ng/mL, time to reach peak concentration increased from 0.75 ± 0.35 to 4 h, and half-life increased from 4.61 ± 0.44 to 29.1 ± 1.30 h.	92
Osteoarthritis	Lornoxicam/MicroRNA-140 (suppress osteoarthritis inflammation) Loading not mentioned	EYL:Chol: C18H37N:Tween-80 Weight ratio: 10.7:1.8:1:5:1.25	Female Wistar rats with osteoarthritis induced by intra-articular papain injection.	The cationic liposomes protected microRNA-140 from RNase degradation for 24 h. Joint width growth differed significantly between microRNA and Lornoxicam alone vs the cationic formulation (approx. 0.5 cm). By 8 wk, the formulation nearly matched the negative control.	29

6-MNA, 6-methoxy-2-naphthylacetic acid; C18H37N, stearylamine; CFA, complete Freund adjuvant; Chol, cholesterol; CMC, carboxymethyl cellulose sodium salt; DLPC, 1,2-dilauroyl-sn-glycero-3-phosphocholine; DSPC, 1,2-distearoyl-sn-glycero-3-phosphocholine; DSPG, 1,2-distearoyl-sn-glycero-3-phospho-(1′-rac-glycerol); EPC, l-α-phosphatidylcholine; EYC, egg yolk lecithin; HSPC, hydro soy phosphatidylcholine; LUV, large unilamellar vesicle; MLV, multilamellar vesicles; mPEG-DSPE - 1,2-distearoyl-sn-glycero-3-phosphoethanolamine-N-[methoxy(polyethylene glycol)]; MPEG-DSPE2000 - sodium; [(2R)-2,3-di(octadecanoyloxy)propyl] 2-(2-methoxyethoxycarbonylamino)ethyl phosphate.

Liposomes, composed of phospholipids and often Chol, can be surface-modified with PEGylation to enhance their biological properties (Fig. 2B). Chol improves membrane stability, which is crucial for maintaining the integrity of the systems in complex biological environments, whereas PEGylation increases circulation time by reducing opsonisation and immune clearance.^33^ However, PEGylation may interfere with cellular uptake and reduce drug loading capacity^76^

Liposomes can also be classified into unilamellar vesicles (ULVs) or multilamellar vesicles (MLVs) (Fig. 2E). Unilamellar vesicles, with a single lipid bilayer, offer higher encapsulation efficiency for hydrophilic drugs and more uniform size distribution, allowing better control over drug release kinetics.^5^ MLVs, consisting of multiple concentric lipid bilayers, provide longer drug release and higher encapsulation efficiency for lipophilic drugs.^61^ Unilamellar vesicles offer higher encapsulation efficiency for hydrophilic drugs and a more uniform size distribution. In comparison, MLVs often have lower encapsulation efficiency for hydrophilic drugs and a more heterogeneous size distribution.^54^ Multilamellar vesicles might be preferred for sustained-release formulations, whereas ULVs could be more suitable when precise control and surface properties are crucial.^61^

### 4.2. Parental delivery

Parental drug delivery systems have seen significant advancements through various liposomal formulations. PEGylated liposomes have been extensively researched, and they are particularly useful for parental drug delivery. PEGylation can increase the stability of the vesicles in biological fluids, such as blood, which results in longer circulation times and more significant accumulation at the site of action.^33,77^ The studies by Gómez-Murcia et al.,^26^ Vivek et al.,^85^ Yin et al.,^89^ and Mennini et al.^55^ demonstrate the diverse therapeutic advantages of PEGylated liposomal formulations using in vivo models.

Gómez-Murcia et al.^26^ and Yin et al.^89^ developed a dual approach of PEGylated liposomes that also contain cholesterol (Chol) in the membrane. Gómez-Murcia et al.^26^ were interested in developing a nonaddictive replacement for intraperitoneal morphine. By encapsulating morphine, its release is controlled, and its possible abuse is reduced. Their research implemented both MLVs and LUVs; despite the higher drug entrapment by MLVs they also showed a quick initial release. By contrast, PEGylated LUVs exhibited higher stability and sustained morphine release. This effect was reflected in their behavioural assessments in mice, where both MLVs and LUVs conferred prolonged analgesic effects. However, LUVs suggested a significant reduction in addiction-related effects compared with free morphine and MLVs.

Yin et al.^89^ focused on a more localised parental approach, developing a sciatic nerve blocker by peri-sciatic injection. Their formulation encapsulated N-methyl bromide of lidocaine (QX-314), a sodium channel blocker with poor lipid tissue penetration. Specifically, their study aimed to discern the effects of cationic, anionic, and neutral PEGylated liposomes on the permeation and effect on QX-314. When applying these vesicles using a peri-sciatic injection in Sprague-Dawley rats, the anionic liposomes significantly prolonged the sensory and motor blockade. By contrast, the cationic and neutral liposomes showed minimal effects on extending these effects compared with pure QX-314. The study concluded that anionic liposomes provided superior performance in enhancing nerve block effects because of their favourable electrostatic interactions with the positively charged QX-314, which facilitated its cellular entry and retention at action sites around the nerve.

Vivek et al.^85^ and Mennini et al.^50^ did not use Chol for their liposomes because their drugs were delivered directly into the bloodstream rather than a localised delivery. However, just like Gómez-Murcia et al.^26^ and Yin et al.,^89^ the liposomes were PEGylated with monomethoxy polyethyleneglycol 2000-distearoyl phosphatidyl-ethanolamine (mPEG-DSPE) for the formation of the vesicles. Vivek et al.^85^ encapsulated celecoxib, a hydrophobic drug, within the lipid bilayer, potentially improving its solubility and bioavailability. However, Mennini et al.^50^ presented a novel approach by encapsulating opiorphin, a short-action pentapeptide whose encapsulation efficiency and stability within liposomes could be more challenging compared with small molecule drugs like celecoxib. The injectable nature of these liposomes allows for an extended drug release in the bloodstream, reducing the frequent administration of the drug.

Encapsulation efficiencies in the study by Vivek et al.^85^ and Mennini et al.^50^ were different, largely because of the physicochemical properties of their encapsulated drugs. Vivek et al. achieved a high encapsulation efficiency of 96% with celecoxib, a small hydrophobic molecule. By contrast, Mennini et al. reported a lower, yet still significant, encapsulation efficiency of 70% for opiorphin, a larger, hydrophilic peptide molecule. The difference in encapsulation efficiency between these 2 formulations underscores the impact of drug properties on liposomal design. Opiorphin, being water-soluble, was encapsulated within the aqueous core of the liposomes, whereas celecoxib integrated into the lipid membrane itself. Despite these differences, both formulations exhibited significantly enhanced analgesic effects compared with their unencapsulated counterparts at equivalent dosages, with increased intensity and prolonged duration of action. Each approach offers unique advantages in pain management. The celecoxib-loaded liposomes show promise for providing sustained anti-inflammatory effects with potentially reduced systemic side effects, making them particularly suitable for chronic inflammatory pain conditions. Conversely, the opiorphin-loaded liposomes represent an innovative strategy to enhance endogenous pain control mechanisms, potentially offering a safer alternative to traditional opioid analgesics.

Building on the foundation of PEGylated liposomes, researchers have explored more specialised formulations to address specific challenges in parental delivery. One such innovation is the development of externally triggerable nanocarriers, which offer precise control over drug release. The application of externally triggerable nanocarriers could provide better control over the timing and location of drug release, enhancing the therapeutic effect while diminishing side effects. One such trigger is light because of the ease of control of wavelength, power, and duration. Upon irradiation with light, photosensitisers can produce reactive oxygen species (ROS) (ie, singlet oxygen) that cause the peroxidation of unsaturated lipids. This destabilises the liposome integrity, allowing the release of its contents. Rwei et al.^68^ integrated the near-infrared (NIR) photosensitiser 1,4,8,11,15,18,22,25-octabutoxyphthalocyaninato palladium(II), PdPC(OBu)8 (abbreviated PS) into their liposomes (using membrane encapsulation) loaded with the drug tetrodotoxin (TTX), which blocks voltage-gated sodium channels. The other lipids that made up the liposome were 1,2-dilinoleoyl-*sn*-glycero-3-phosphocholine (DLPC), an unsaturated lipid that contributes to the membrane fluidity improving drug encapsulation, 1,2-distearoyl-*sn*-glycero-3-phosphocholine (DSPC), a saturated lipid provides structural stability, and 1,2-distearoyl-*sn*-glycero-3-phosphatidylglycerol (DSPG), which imparts an anionic charge to the liposomes.

Rwei et al.^68^ induced and reversed the nerve block up to 4 times from a single administration in rats. Notably, the near-infrared light trigger (730 nm, 500 mW/cm^2^) effectively penetrated tissue to depths of about 1 to 2 cm without causing thermal damage, enabling noninvasive control of the nerve block. The liposomes demonstrated excellent stability, maintaining their integrity for up to 6 days postinjection, a significant improvement over traditional local anaesthetic formulations. Furthermore, the photo-triggerable liposomes exhibited a favourable safety profile, with no signs of nerve damage or systemic toxicity observed at the effective dose (0.5 mg/kg of TTX). This approach enhanced the precision of analgesia and potentially reduced the total amount of anaesthetic required, as evidenced by the ability to achieve effective nerve blocks with lower doses than conventional methods.

Although these advanced liposomal systems are promising in preclinical studies, translating such technologies into clinical practice remains challenging. Only one liposomal formulation for parenteral delivery of pain management drugs has progressed to clinical trials, highlighting the gap between laboratory innovation and clinical application. This formulation, developed by Grant et al.,^24^ uses MLVs to enhance the release rate of bupivacaine. Through freeze-thaw cycles, the researchers created MLVs using soy phosphatidylcholine and cholesterol. These MLVs were then loaded with bupivacaine using remote loading using a sulfate gradient. In a small clinical trial, 6 subjects received intradermal injections of the formulation in their lower backs. Their sensitisation in the treated area was evaluated using a spinal needle at different intervals. Results demonstrated that the liposomes provided sustained and prolonged dermal analgesia compared with standard bupivacaine. This formulation, known as Probudur, is undergoing clinical trials as an ultra-long-acting local anaesthetic for postoperative pain.^83^

### 4.3. Topical delivery

Liposomes have been explored with other delivery carriers, such as hydrogels. Hydrogels are 3-dimensional networks of hydrophilic polymers that can absorb and retain large amounts of water, resulting in a viscoelastic, gel-like consistency.^42^ Abd El-Alim et al.^1^ and Iwaszkiewicz and Hua^35^ developed a liposome–hydrogel system for transdermal delivery in male Wistar rats. The hydrogels work as a matrix to form a depot of liposomes on the skin surface to control their release. Drug-loaded liposomes are engineered to be flexible to deform and pass through small pores, such as those found in a hydrogel matrix and the initial skin barrier. This can be achieved using lipids such as l-α-lecithin from soybean oil and l-α-phosphatidylcholine.

Iwaszkiewicz and Hua^35^ developed a loperamide-based formulation capable of delivering to the dermis's free nerve endings. Loperamide binds to cellular membranes rather than readily penetrating the skin, making it unsuitable for topical delivery. Therefore, the l-α-phosphatidylcholine liposomes overcame this shortcoming by delivering this drug more effectively to its target on the skin. The formulation used a Carbopol gel base at 0.5% to 1% (wt/wt) concentration, which provided ideal pH and rheological characteristics for topical application while aiding liposome stability. The study demonstrated significant efficacy of the loperamide HCl–encapsulated liposomal gel in the complete Freund adjuvant (CFA) rat model of acute peripheral inflammatory pain. The formulation achieved a significant increase in paw pressure threshold (PPT) over a 48-hour study period compared with baseline, indicating substantial analgesic efficacy. This effect was reversible with naloxone methiodide, confirming an opioid receptor–dependent mechanism. Notably, the liposomal formulation showed superior efficacy to free loperamide gel, suggesting successful dermal delivery of the drug to peripheral opioid receptors. The liposomal formulation achieved this with twice-daily application, compared with 3 times daily for diclofenac, potentially offering improved patient compliance. Moreover, the liposomal formulation showed anti-inflammatory effects not observed with diclofenac in this study, highlighting its potential clinical significance in managing hyperalgesia and inflammation associated with peripheral inflammatory pain.

With this approach, Abd El-Alim et al.^1^ encapsulated diflunisal, a common oral drug, enabling its use as a topical analgesic. In the antinociceptive activity evaluation, their formulation showed a delayed onset of action. It only exhibited a significant decrease in flinches from 4 to 8 hours after application, compared with the plain diflunisal hydrogel. This delayed effect can be attributed to the structural limitations of conventional liposomes in transdermal delivery. The authors note, “Due to a lack of deformability, liposomes do not penetrate efficiently into deep skin layers and are mostly stopped in the epidermis upper layers.” This poor penetration ability results in the drug accumulating in the upper skin layers, leading to a slower release into the highly vascularised dermis. Despite these limitations, it is important to note that the liposomal formulation still showed improvements over the plain duflinasal. This suggests that even with their penetration limitations, liposomes can enhance drug delivery to some extent, possibly by increasing drug solubility, protecting the drug from degradation, and providing sustained release.

### 4.4. Intracellular delivery

As mentioned previously, one possible strategy for intracellular delivery is to include cationic lipids, eg, 1,2-dioleoyl-3-trimethylammonium-propane (DOTAP), as a component of the liposomal membrane.^75^ DOTAP is a lipid with a positive charge in the trimethylammonium group. This facilitates nonspecific electrostatic interaction with the negatively charged cell surface, enhancing cell uptake by endocytosis.^20^ Cationic lipids are a well-established strategy for formulating nucleic acid therapeutics into liposomes for delivery.

He et al.^29^ explored this approach to develop lornoxicam/miRNA-140 co-loaded cationic liposomes for osteoarthritis. The synergistic action between the lornoxicam (an NSAID to reduce inflammation), the miRNA-140 (to reduce arthritic damage), and the liposome itself, which enables intracellular delivery of the miRNA, provided a comprehensive approach to osteoarthritis treatment, addressing both symptoms and underlying causes. Compared with other treatments, including naked miRNA-140 or miRNA-coated liposomes (without the drug), their formulation demonstrated superior efficacy in reducing knee swelling, preserving cartilage structure, and minimizing synovial inflammation.

Building on the concept of improving drug retention and targeted delivery, Pawar et al.^63^ developed a liposomal drug to deliver 6-methoxy naphthalene acetic acid (6-MNA), a nonselective COX inhibitor, for treating knee osteoarthritis. Because cartilage tissue contains negatively charged sugars, developing a positively charged liposome would improve drug residence time in the delivery site. Their formulation consisted of hydrogenated soy phosphatidylcholine (HSPC), DOTAP, Chol, and 6-MNA-DSPE-Na double salt (synthesised in-house as the drug-fortified agent). The synthesis of 6-MNA-DSPE-Na double salt was achieved through the reaction between the basic amino group of 1,2-distearoyl-*sn*-glycerol-3-phosphoethanolamine (DSPE)-Na and the carboxylic acid functional group present in 6-MNA. This ionic interaction allows for a sustained and controlled release from the vesicles. 6-MNA was also encapsulated in the membrane of the liposomes. Tests in a complete Freund adjuvant (CFA) rat model showed that this formulation improved therapeutic efficacy because of the coupled effect of longer vesicle retention obtained with DOTAP and sustained release from the liposome (fortifying agent).

Although both strategies aim to improve the therapeutic efficacy of NSAIDs in arthritis treatment, the first study's approach offers potential advantages regarding joint cavity retention and the possibility of synergistic effects between the free drug and its lipid-salt form. However, the simpler formulation in the second study might offer benefits in ease of preparation and potentially broader applicability across different NSAIDs. By contrasting these 2 methods, we can better understand the strengths and limitations of various liposomal formulations for intracellular delivery in pain management.

### 4.5. Other administration routes and unconventional drugs

Liposomal technologies opened the way for unconventional drugs to be considered in the treatment of pain. One example of this was developed by Zhu et al.,^92^ who designed soybean lecithin phosphocholine (PC) vesicles loaded with capsaicin (the active alkaloid of chilli peppers) as the active pharmaceutical ingredient. Oral administration of capsaicin produces irritation in the gastrointestinal tract. Encapsulation into liposomes negated this effect in rats. This report is limited to a toxicological in vitro assessment of these formulations; their effects on pain treatment in vivo are yet to be reported. Shin et al.^72^ encapsulated another unconventional agent for the treatment of pain, KT109, which is an inhibitor of the diacylglycerol lipase-beta (DAGLβ) enzyme, loaded into DSPC:DOPE:PEG_2000_PE vesicles, KT109 inactivates DAGLβ in macrophages, resulting in an anti-inflammatory effect and thereby reducing pain in injured tissues. Notably, the liposome formulation includes dioleoyl phosphatidylethanolamine (DOPE), a common fusogenic lipid thought to promote the fusion of the liposomes with the endosomal membrane for efficient delivery. However, the liposomal drug encapsulation for this formulation was significantly low (0.4%), so the authors concluded that it would not be economically viable for clinical translation.

## 5. Polymersomes, ethosomes, and niosomes for pain treatment

### 5.1. Polymersomes

Polymersome technology only started to gain traction and popularity at the turn of the century.^17^ This may, in part, be because of the potential disadvantages of polymer-based vesicles compared with lipid-based systems for pain management therapeutics. Biocompatibility and stability of vesicles are 2 critical factors to consider when developing nanocarriers. Polymersomes have exhibited lower biocompatibility and higher stability than liposomes, which can lead to drug retention for more extended periods of time than required for an effective therapy.^51^ Despite these considerations, one preclinical study has been performed with polymer vesicle formulations for pain management (Table 3).

**Table 3:** Polymersomes and lipo-polymersomes for pain treatment.

Application	Drug encapsulated, efficiency and method	Vesicle composition	Experimental setup (in vivo unless otherwise stated)	Results	Ref
Polymersomes					
Neuroinflammation	Simvastatin ∼100% compared with initial drug Nanoprecipitation method	PEG-PdLLA	Measurement of cytokines NO, TNF-alpha, and IL-6 in BV2 microglial cells.	Simvastatin-loaded polymersomes outperformed the positive control, reducing NO, TNF-α, and IL-6 by 52%, 84%, and 60%, respectively. Free simvastatin reduced NO by 40% and TNF-α by 60%, but had no significant effect on IL-6 compared with the positive control.	49
Ethosomes					
Anti-inflammatory	SPC:Chol:Ethanol 2.5% (wt/vol): 1.5% (wt/vol): 25% (vol/vol)	Paeonol 84.33 ± 1.34% compared with initial drug Ethanol injection	Ex vivo*:* Skin irritation and drug permeation tests conducted	Paeonol ethosomes showed significantly higher skin permeation (135.14 ± 15.2 µg/cm^2^) compared with 25% hydroethanolic solution (52.60 ± 7.90 µg/cm^2^). Ethosome-treated skin did not differ significantly from untreated control.	47
Local anaesthetic	PC (origin not mentioned): Chol	Lidocaine Highest encapsulation of 66.5 ± 4.3% compared with initial drug Ethanol injection	Skin permeation experiments Male Wistar rats	Free lidocaine penetrated skin more effectively than ethosomes. Yet, ethosomes prevented systemic absorption, enabling sustained release	7
Anti-arthritic	PC (phospholipon 90 G): ethanol 2%:30% (wt/vol)	Capsaicinoids extract from *Bhut jolokia* (hottest capsicum)	In vitro*:* Skin permeation. In vivo*:* Adult Wistar CFA-induced paw inflammation	Ethosomes penetrated deeper skin layers compared with a marketed capsaicin formulation and a hydroethanolic solution. Untreated rats had a paw volume of 1.1 ± 0.95 cm^3^. The marketed capsaicin formulation showed 0.87 ± 0.09 cm^3^, whereas the ethosomal formulation exhibited 0.66 ± 0.11 cm^3^ 24 h after CFA injection.	70
Niosomes					
Chronic pain	Tween-20:Chol 11.25 mM:7.5 mM	Ibuprofen 0.82 ± 0.05 mg drug/mL of niosomes Passive encapsulation	CD-1 mice capsaicin-induced paw licking	The niosomal formulation demonstrated a significant antinociceptive effect compared with free ibuprofen, as evidenced by reduced paw licking time during the 6-h observation.	52
Chronic inflammation	Tween-20:Chol See compositions used in the reference	Ibuprofen 0.28 ± 0.04 mg drug/mL of niosomes Lidocaine 8.65 ± 0.04 mg drug/mL of niosome Passive encapsulation	CD-1 mice carrageenan-induced paw licking	Ibuprofen- and lidocaine-loaded niosomes significantly reduced formalin-induced licking activity by nearly half compared with untreated paws in the same timeframe.	67

CFA, complete Freund Adjuvant; Chol, cholesterol; NO, nitric oxide; PCL, poly-ε-caprolactone; PEG-DSPE, poly (ethylene glycol)-distearoylphosphatidylethanolamine; PEG-PdLLA, methoxy-poly (ethylene glycol) poly (d, l-lactide) copolymer; SPC, soybean phosphatidylcholine.

PEG-poly(d, l-lactide) (PdLLA) is a block copolymer that is widely investigated for biomedical applications because of its biocompatibility, biodegradability, and stealth properties (derived from its PEG coating).^10,78^ Manickavasagam and Oyewumi^49^ chose this block copolymer because of the polymersome's enhanced stability compared with liposomes, allowing longer sustained simvastatin delivery. This drug is currently being tested for neuropathic pain. Their formulation demonstrated a sustained release of approximately 88% over 24 hours in vitro, while free simvastatin was fully released within 2 hours (from a dialysis bag). This controlled release outlasted the free simvastatin and resulted in a more significant reduction in inflammatory cytokines (6 times reduction) 24 hours after inflammatory activation induced by lipopolysaccharides on BV2 microglial cells. Additional in vivo experiments would provide valuable insights into the therapeutic effectiveness of the sustained simvastatin release in a complex biological environment, including its impact on neuropathic pain models and potential side effects.

### 5.2. Ethosomes

One of the main disadvantages of transdermal drug delivery is the poor penetration of most drugs into the human skin. The stratum corneum (SC) is the principal barrier that protects the skin (outer layer) from exogenous substances entering the body.^82^ Many methodologies have been developed to increase skin permeation. However, these approaches mainly involve chemical enhancers that may cause damage and reduce skin function. Ethanol is known to be an effective permeation enhancer and as such, ethosomes, which contain 20% to 45% ethanol, can penetrate through pores that are much smaller than their diameter. Current ethosomes for pain management are summarised in Table 3.

Because of the high skin permeation ethosomes may offer, they have been investigated for the formulation of drugs for transdermal pain therapy. Babaie et al.^7^ showed the potential of this technology by optimising a PC ethosome loaded with lidocaine. They do not clearly state the origin of the PC used for their experiments, but it slowly released the drug in a systemic drug absorption simulator (in vitro 2.5% release after 24 hours). In comparison, a lidocaine hydroethanolic solution (control) had a better permeation (∼14% after 24 hours); nevertheless, by doing so it became more susceptible to systemic absorption. Their in vivo experiments demonstrated that ethosomes effectively permeated rat skin better than commercially available lidocaine. However, their research was limited to verifying the permeation, and hence, the effectiveness of the ethosomal formulation remains unknown.

Ma et al.^47^ and Sarwa et al.^70^ encapsulated 2 highly skin-irritable drugs, paeonol and capsaicinoids. Both substances are promising painkillers. Encapsulation in an ethosome could potentially avoid skin irritation and enhance permeation profiles. Ma et al. did not test the therapeutic efficacy of their ethosomal formulation. Instead, their study assessed skin irritation and drug permeation through ex vivo models. Their ethosome was composed of soybean PC and Chol with an ethanol content of 25% (vol/vol). The formulation had a higher skin permeation than the free drug hydroethanolic solution. Similarly, cross-sections of ethosome-treated skin showed no significant difference in the level of irritation compared with the untreated controls.

Sarwa et al. (2014) measured the skin irritation, drug permeation profiles and the efficacy of their capsaicinoid-loaded ethosome. Encapsulating capsaicinoids in ethosomes reduced the initial irritation effect, improving in vivo acceptability. This was attributed to the smoothing properties of phosphatidylcholine and the cooling effect of ethanol, which together minimised skin irritation typically caused by capsaicinoids. The study confirmed the potential of capsaicinoid-loaded ethosomes for treating inflammation and pain associated with arthritis. The flexible nature of ethosomes allowed them to penetrate deeper into the skin, making them more effective than traditional topical formulations. The localized administration of capsaicinoid ethosomal nanovesicles could inhibit peripheral activation of primary sensory afferent neurons, providing effective pain and inflammation relief with minimal systemic side effects.

### 5.3. Niosomes

Niosomes are a type of vesicle whose core components are nonionic surfactants and cholesterol. A nonionic surfactant from a fully ionised functional group in the hydrophilic head has no net charge. Niosomes have a long shelf-life and high stability and are safe for biomedicines. They are a relatively new technology, and little is known about their biophysical properties.^3,65^ Table 2 presents a summary of recently developed niosomes.

Marzoli et al. (2019) investigated the potential of an ibuprofen-loaded Tween-20:Chol niosome in a mouse model. Tween-20-glycine is a nonionic detergent and emulsifier. The loaded niosomes were stable for at least 3 months when stored at 4°C and were similar to regular liposomes in that they were spherical in shape. After the induction of an inflammation response in the mouse paw with capsaicin, niosomes showed a statistically significant antinociceptive effect compared with free drug. The researchers recorded the time the mice spent licking the inflamed area for these measurements. Rinaldi et al. (2017) prepared niosomes for delivering ibuprofen or lidocaine using Tween-20-glycine and Chol in different molar ratios. Their research showed that niosomes with lidocaine had higher encapsulation efficiency and stability compared with those with ibuprofen. The difference in encapsulation efficiency was attributed to electrostatic repulsion between ibuprofen and the niosome surface. Lidocaine-encapsulated niosomes reduced the paw inflammation volume and the number of writhes in mice, indicating a reduction in pain compared with free lidocaine.

Tween 20-gly in the niosomal formulation also offers controlled release and stability advantages. These vesicles remain stable under neutral pH conditions but undergo destabilisation in acidic environments, which prolongs drug activity at the target site, protects the drug from premature degradation, and enhances the vesicle's ability to release drugs in acidic environments typical of inflammation. By targeting drug delivery and controlling the drug release, Tween 20-Gly niosomes can minimise systemic exposure and reduce potential side effects, making them suitable for various therapeutic applications.

## 6. Exparel: analysing clinical research after food and drug administration approval

The development of vesicle-based nanomedicines, in general, is moving at a fast pace, yet the same challenges remain when translating research towards the clinical market. The first challenge is financial; if a newly developed vesicle drug does not compensate for the cost of its mass production and an overall reduction in healthcare expenses compared with conventional therapies, then it is deemed not viable and unattractive for capital investors.^31^ Second, including multiple excipients in advanced vesicle formulations increases the challenges and complexity of the scalability of the manufacturing process. Consequently, manufacturers may not be ready to produce or unable to ensure the final product's quality.^71^ The simpler the formulation, the easier it is to mass produce.

Despite these obstacles, one vesicle formulation has gained approval for treating pain: Exparel. Exparel was approved by the U.S. Food and Drug Administration (FDA) in 2011 and was developed by Pacira Pharmaceuticals, San Diego, CA. It consists of microscopic, spherical lipid-based particles (known as the DepoFoam delivery system) composed of a honeycomb of numerous, nonconcentric, internal aqueous chambers containing bupivacaine.^50^ Each chamber is separated from adjacent chambers by lipid membranes composed of synthetic analogues of naturally existing lipids (DOPC, DPPG, cholesterol, and triolein). This distinctive feature sets it apart from conventional ULVs, with a single aqueous chamber, and MLVs, which exhibit an onion-like structure. The loading efficiency of bupivacaine is generally less than 10 wt%.^50^ At first, it was named Skye 0402, changing to DepoFoam bupivacaine, and finally, Exparel. For its approval, the FDA reviewed 22 studies focused on haemorrhoidectomy and bunionectomy procedures, concluding that Exparel reduced pain intensity compared with the placebo group for up to one day. After the first 24 hours, there was no difference between Exparel and other treatments.^73^

Multiple studies have clinically tested Exparel against different analgesics with mixed results (Table 4). Six of 12 studies in Table 4 conclude that Exparel did not have a significant benefit over conventional analgesic regimes in applications such as postoperative abdominal pain,^14,21,41^ breast reconstruction,^28^ shoulder arthroplasty,^2^ total knee arthroplasty,^86^ and symptomatic irreversible pulpitis.^9^ One study in Table 4 was inconclusive.^14^ These studies did not see an improvement in the pain relief capabilities of the formulation compared with the regular unencapsulated bupivacaine or commonly used analgesic regimes. However, 5 of the studies in Table 4 did report patient benefit, highlighting the clinical potential of long-acting analgesic formulations. Therefore, Exparel has opened the way for the approval of other, potentially improved, vesicle-based technologies in pain management. For example, Virpax Pharmaceuticals Inc. have recently announced the results of dose escalation studies of Probudur (liposomal hydrogel bupivacaine) in rat models compared with the performance of Exparel: they claim that Probudur has a 3 to 5 times longer duration of efficacy compared with Exparel. However, it should be noted that, to our knowledge, these studies have not yet been published in a peer-reviewed format.^84^ These results will support Probudur's application to be filed as an Investigational New Drug, with the aspiration for first-in-human trials to begin in 2024.

**Table 4:** Studies testing exparel with common analgesic drugs and their outcomes.

Application	Study setup	Outcomes measured	Results	Funding source	Ref.
Breast reconstruction	N = 44 1:1 random allocation LB:plain bupivacaine (PB) Mean age: 49 ± 10 Single surgeon, patient blinded to the anaesthetic used.	Primary: total intraoperative and postoperative opioid consumption. Secondary: quality of recovery scores	There was not statistically or clinically significant difference in the quality of recovery, pain scores, or length of stay between liposomal bupivacaine and conventional bupivacaine.	The Foundation for Barnes-Jewish Hospital.	40
Postoperative pain control	N = 924; n = 356 (LB) and n = 568 (PB) Age: 36 wk–16 y 1:2 match (LB:PB) Imbalance assessed using absolute standardised difference (ASD ≥0.13 considered as imbalanced) Two blinded researchers determined the outcomes from medical data (retrospective review)	Signs of LAST and major complications after surgery	Liposomal or plain bupivacaine was administered during surgery. There were a few cases of LAST in both groups, but they could be related to the administration. Given the limitations of the medical records analysed, it was not possible to provide a clear conclusion. The authors propose a larger cohort with adequate data acquisition from patients.	PACIRA Pharmaceuticals, Inc.	14
Perioperative pain control	N = 191; n = 94 (LB) and n = 97 (PB) Mean age: 43.7 Retrospective chart review	Primary: total analgesic consumption Secondary: quality of recovery	Preoperative transversus abdominis plane (TAP) block with LB significantly decreased the use of opioid analgesics. However, a more extensive prospective study is needed to validate the results further.	Not reported, authors declare no conflicts of interest	57
Colon and rectal surgery	N = 179; n = 92 (LB) and n = 87 (epidural analgesia) Mean age: 62 1:1 random allocation. Single-institution, parallel-group, open-labelled randomised controlled trial.	Primary: numeric pain scale and overall benefit of analgesia score. Secondary: postoperative complications, opioid consumption, and costs.	The study did not show a significant difference in the numeric pain scale and overall benefit of analgesia score between the groups treated with epidural analgesia and LB. Patients treated with epidural analgesia had higher numeric pain scores on the day of the surgery compared with patients treated with LB. Later in the postoperative period, the relationship was inversed.	Not reported, authors declare no conflicts of interest.	21
TKA	N = 119; n = 40 (LB), n = 41 (PB + morphine), and n = 38 (PB) Mean age: 69 Prospective, multicentre, double-blind randomised trial. Five surgeons at 2 centres performed the surgeries.	Primary: Pain scores and total narcotics use. Secondary: ambulation, quality of recovery.	Mean pain scores in the LB group were lower than the periarticular injection of ropivacaine at 6 h and 12 h after surgery. The investigators report that the differences are near the lower end of minimal clinically significant differences and may only represent a slight clinical improvement.	The authors report research support from PACIRA pharmaceuticals, Inc., and Zimmer Biomet (Pacira Pharmaceuticals)	8
Midurethral slings	N = 109 n = 54 (LB) n = 55 (placebo) Mean age: 52 Randomised, placebo-controlled trial. Only patients were blinded to the treatment.	Primary: visual analog pain scores (VAS) after discharge. Secondary: narcotic consumption	The pain scores were lower for the groups that took the liposomal bupivacaine; however, the pain was low in both the placebo group and plain bupivacaine. Participants who received the liposomal formulation were less likely to use narcotics on postoperative day 2.	Not reported, authors declare no conflicts of interest.	53
Shoulder arthroplasty	N = 83; n = 36 (LB) and n = 47 (IINB) Age >18 Prospective, randomised controlled trial.	Primary: VAS and opioid consumption. Secondary: postsurgery complications, length of stay	The immediate postoperative pain relief was better with an indwelling interscalene nerve block than with liposomal bupivacaine. Opiate consumption was significantly higher in patients with the liposomal treatment in day 0 and for the total hospital stay.	Not reported, authors declare no conflicts of interest.	2
Symptomatic irreversible pulpitis	N = 95; n = 47 (LB) n = 48 (PB) Mean age: 33 Single-blind controlled study.	Primary: Tissue numbness and postoperative pain Secondary: Total narcotic consumption	LB did not reduce the pain to manageable clinical levels. The authors do not recommend the use of Exparel to control postoperative pain in patients with symptomatic irreversible pulpitis.	Not reported, authors declare no conflicts of interest.	9
Colorectal surgery	N = 57; n = 27 (LB) and n = 30 (PB) Mean age: 67 Double-blinded, prospective, randomised controlled trial.	Primary: total opioid consumption postsurgery Secondary: length of stay, quality of recovery	There was no significant difference between the group treated with LB and bupivacaine HCl in the number of opioids used orally or intravenously in the postoperative period. Hence, LB does not provide any added benefit over conventional bupivacaine after colorectal surgery.	Not reported, authors declare no conflicts of interest.	41
TKA	N = 533; n = 267 (LB + PB) and n = 266 (PB) Mean age: 69 Multicentre (11 centres), patient-blinded, pragmatic, randomised clinical trial	Primary: quality of recovery and VAS (6–72 h postsurgery) Secondary: Quality of recovery and VAS at day of surgery	Patients treated with LB walked farther on the day of surgery and were more likely to be discharged within 2 days than patients treated with a combination of control drugs. The direct hospital cost per patient was lower with Exparel.	The National Institute for Health Research (NIHR) and investigation of medicinal product from PACIRA pharmaceuticals.	39
TKA	N = 120; n = 55 (LB) and n = 65 (control) Mean age: 67 Single-centre retrospective cohort	Primary: pain scores from surgery to 48 h Secondary: mean total opioid consumption, quality of recovery	There is no substantial pain relief when using LB for patients undergoing TKA compared with using ropivacaine infusions.	Not reported, conflict of interest not reported.	86
Postsurgical pain management	N = 911; n = 505 (LB) and n = 406 (PB) TKA, hemorrhoidectomy, breast augmentation, and bunionectomy Mean age: 50 Double-blind multimodal study. Efficacy and safety data from 9 studies were pooled and analysed	Primary: pain intensity scores from at least 72 h after surgery. Secondary: first opoid use time, total consumption, and quality of recovery.	The mean cumulative pain score was significantly lower when using LB compared with bupivacaine HCl, resulting in less opioid consumption and fewer opioid-related adverse events.	PACIRA pharmaceuticals, Inc.	15

IINB, indwelling interscalene nerve block; LAST, local anaesthetic systemic toxicity; LB, liposomal bupivacaine; TKA, total knee arthroplasty.

## 7. Conclusion and future perspectives

Exciting breakthroughs in vesicle engineering can potentially improve pain management technologies in a clinical setting. Vesicles can offer increased benefits in pain treatment. The tunability of incorporating different molecules in the membrane or lumen is currently being exploited to create new drug-release systems that result in superior control over the temporal and spatial bioavailability of pain-reducing drugs. A more targeted and long-acting delivery approach using vesicle formulations has the potential to reduce side effects and addiction-related issues caused by opioids. Emerging nanomedicines like Exparel, which claims a more prolonged effect than free bupivacaine with reduced side effects, exemplify the transformative potential of vesicle-based therapies. These advancements enable faster recovery and return to patient societal productivity, reducing economic costs. However, despite the promising improvements in these technologies, there are barriers to their broader clinical adoption, highlighting the need for continued research and development.

Vesicles offer a versatile platform for the delivery of single therapeutic agents and the codelivery of multiple therapeutics, which can synergistically target different aspects of pain pathways.^88^ For example, vesicles can be loaded with both opioid analgesics for immediate pain relief and anti-inflammatory agents to mitigate inflammation, thereby potentially reducing the degree of opioid receptor desensitisation and the consequent development of tolerance over time.^30^ A novel combination using this strategy is the codelivery of small interfering RNAs (siRNAs) targeting pain-associated genes along with analgesic peptides or growth factors to provide long-lasting pain relief while addressing the underlying causes of pain. This strategy, which has shown promise in treating conditions such as cancer,^91^ necessitates efficient intracellular delivery to exert its effects.^19^

An emerging avenue for enhancing intracellular delivery is through engineering small extracellular vesicles (sEVs), specialised vesicles naturally generated by cells which work as a form of communication between them.^90^ Although artificial vesicles can be tailored to enhance intracellular delivery through surface modifications, sEVs could contain proteins, nucleic acids, and lipids, which reflect the cell of origin, increasing its biocompatibility and intracellular delivery capacities.^45^ These characteristics have attracted attention to treat painful neuropathies because of their natural role in neuroregeneration and protection^37^ and their capacity to traverse the blood–brain barrier and subsequently infiltrate other nervous system regions.^59^ Nevertheless, the scalability and reproducibility of sEVs remain a significant challenge. Current methods for isolating and purifying exosomes often lead to variable results, making them a crucial obstacle in translation to the clinical market.^59,90^

Integrating diagnostic molecules within vesicles could offer a paradigm shift in precision medicine, enabling real-time pharmacokinetic and pharmacodynamic monitoring. This approach optimises drug delivery, distribution, and targeting, ultimately enhancing therapeutic outcomes. Engineered vesicles can simultaneously encapsulate imaging agents and therapeutic molecules, allowing for noninvasive tracking of treatment efficacy and timely adjustments to therapy regimens.^58^ The synergistic combination of diagnostics and therapeutics, often referred to as theranostics, has the potential to revolutionise chronic pain management by providing a continuous feedback mechanism. This ensures optimal drug dosing while minimising the risk of adverse effects.

Furthermore, targeting immune cells, particularly macrophages, with vesicle-based therapeutics represents a promising strategy in chronic pain management, given their pivotal role in mediating neuro-immune interactions. Macrophages are key orchestrators of the inflammatory response and significantly influence nociception and pain progression.^36^ Vesicles engineered to deliver anti-inflammatory agents or RNA interference tools directly to macrophages can modulate their phenotype and activity, attenuating inflammation and associated pain. This targeted approach can potentially break the cycle of chronic pain by addressing the underlying immune dysregulation that often perpetuates it.

In conclusion, the future of pain management lies in the continued advancement and translation of vesicle-based technologies. By overcoming current challenges and leveraging the versatility of vesicles, researchers can develop novel therapeutic strategies that promise improved outcomes and enhanced quality of life for patients suffering from acute and chronic pain conditions.

## Disclosures

The authors have no conflict of interest to declare.
